# Association of physical activity with socio-economic status and chronic disease in older adults in China: cross-sectional findings from the survey of CLASS 2020 after the outbreak of COVID-19

**DOI:** 10.1186/s12889-023-17492-9

**Published:** 2024-01-02

**Authors:** Yi Li, Mingyuan Cui, Yiqun Pang, Bing Zhan, Xiaotian Li, Qiurui Wang, Fang Chen, Zhixiong Zhou, Qingzhu Yang

**Affiliations:** 1 Capital University of Physical Education and Sports, Beijing, 100000 China; 2 Southwest Petroleum University, Chengdu, 610000 China

**Keywords:** Physical activity, Socio-economic, Association, Older adults

## Abstract

**Background:**

In 2021, China had a population of 264·01 million individuals over the age of 60, indicating a high prevalence of chronic diseases. Among older adults, physical inactivity (PI) is a significant risk factor for chronic diseases. However, few studies have been conducted on the correlation of physical activity (PA) with the economic status, geography and chronic disease risks in Chinese elderly. The objectives of this study were to better understand the distribution of PA among older adults in China and its relationship with economic status, geography, and chronic disease risks.

**Methods:**

This study utilized data from the China Longitudinal Aging Social Survey (CLASS) in 2020, post-COVID-19. The study employed a stratified, multistage, probabilistic sampling approach and included 11,396 adults over the age of 59 from 28 provinces in China. Data on demographics, the duration and intensity of PA, history of diseases and personalized factors influencing PA were collected via structured interviews by researchers. In this study, we conducted a comprehensive analysis, employing a range of statistical methods including descriptive analysis, Wilcoxon rank-sum tests, Bayesian networks, and chi-square tests.

**Results:**

The prevalence of PI among older adults over 59 in China is 28·82%. Significant regional differences were observed in the duration of PA at different intensities. Older adults residing in more economically developed areas were more likely to engage in moderate-to-vigorous physical activity (MVPA) and exhibited longer sedentary behavior. Economic status and urban-rural disparities consistently emerged as direct influential factors across all intensity types. Chronic disease risks were significantly lower in active older adults compared to inactive ones. Lack of social guidance, family support, and personal inclination towards sedentary behavior were the main personalized factors affecting PA among older adults, and these factors could be relatively easily modified.

**Conclusions:**

Economic status, geography, and living areas (urban and rural) significantly influenced the distribution of physical activities in China. Particularly, economic status and living areas acted as direct factors. Older adults reaching the recommended standards for PA had significantly lower chronic disease risks, highlighting the importance of improving personalized factors which are crucial for promoting PA.

**Supplementary Information:**

The online version contains supplementary material available at 10.1186/s12889-023-17492-9.

## Introduction

The population of older adults aged 60 years and above in China reached 264.01 million in 2021, accounting for 18.7% of the total population, highlighting the increasing prevalence of chronic diseases among this demographic [1, 2]. Approximately 180 million older adults suffer from chronic diseases [3, 4], with over 75% of them being diagnosed with one or more chronic conditions [4, 5]. Additionally, there are around 40 million older adults who are disabled or partially disabled [4, 6–8]. Numerous studies have demonstrated that regular PA can effectively reduce the risk of cardiovascular disease [9], osteoporosis [10–12], diabetes [13, 14], falls [15, 16], as well as prevent depression [17, 18], dementia [19, 20], anxiety disorders [21, 22], and other mental illnesses [23, 24]. Meanwhile, it has been proven that regular participation in social activities is significantly correlated with survival time [25]. PI is considered the fourth leading cause of death worldwide and is a major risk factor for non-communicable diseases (NCDs) [26, 27].

Throughout the COVID-19 pandemic, numerous countries implemented essential public health measures, such as home isolation and social distancing, to mitigate the spread of the coronavirus. These measures have had a substantial impact on the PA of older adults. Research conducted across various nations has consistently shown a significant decline in the PA levels of older adults, which could potentially result in more adverse health outcomes, including feelings of isolation, depression, and anxiety disorders [28–33]. For instance, a cross-sectional study from Hong Kong conducted a random telephone survey on the level of PA of adults from May to June 2020. The findings revealed that, before the onset of the COVID-19 pandemic, participants reported an average weekly duration of 1,410 min in sedentary or lying activities, along with an average of 137.6 min per week devoted to MPA. Contrastingly, amid the COVID-19 pandemic, the average weekly duration of sedentary or lying activities surged to 1,897.8 min, while the weekly engagement in MPA dwindled to an average of 108.5 min [34]. Consequently, it is imperative for governments and healthcare institutions to gain a deeper understanding of the needs of older adults by studying and analyzing data from this period. They can provide online fitness resources, psychological support, and remote medical services to assist older adults in maintaining both their physical and mental well-being. Furthermore, due to the varying degrees of impact of COVID-19 in different countries or regions, the strategies employed to combat the pandemic also vary. As a result, the impact of COVID-19 on the PA of older adults also varies to a certain extent. This variance offers an opportunity for more comprehensive research into the PA of older adults. Simultaneously, the cross-sectional data collected in 2020 has facilitated comparisons of disparities in the health and PA levels of older adults across nations. This has contributed to a better understanding of the influence of policies and cultures on the well-being of older adults. Therefore, research conducted during this period holds significant importance.

The Chinese government attaches great importance to addressing the challenges posed by an aging population and is actively promoting the development of the older adults’ care industry [35]. However, the development of relevant policies and action plans for older adults’ health necessitates a comprehensive understanding of the current status of PA among older adults in China and the factors influencing their engagement. Currently, research on accurately quantifying the specific duration of PA distribution among older adults throughout China remains inadequate. Recent studies have predominantly focused on the prevalence of behavioral risk factors such as smoking, heavy drinking, PI, and obesity on a nationwide scale [36, 37]. As a result, it is challenging to obtain a comprehensive understanding of the characteristics of PA distribution and its influencing factors across different provinces among older adults. Moreover, few studies have investigated the correlation between specific durations of PA and the risk of chronic diseases, as well as the personalized factors influencing older adults’ participation in PA. These limitations can result in inadequate allocation of healthcare resources, a shortage of personalized interventions, missed opportunities for early prevention and management of chronic diseases, and hinder the development of a comprehensive national action plan to promote the health of older adults.

Therefore, this study utilized a large sample from the China Longitudinal Aging Social Survey (CLASS) conducted in 2020 to conduct a nationally representative study of PA among older adults aged 59 and above [38]. Firstly, we constructed a map depicting the duration of PA among older adults in China and conducted a comprehensive analysis of influencing factors such as economy status, geography, Human Development Index (HDI) factors and urban-rural living areas. The HDI encompasses three fundamental dimensions: life expectancy, educational attainment, and overall living standards. By employing a specific calculation method, it derives a comprehensive value that serves as a metric for evaluating the economic and social development status of a country or region. Since its inception in 1990, the HDI has gained widespread prominence in the examination of disparities and the assessment of well-being levels within countries and regions [39]. This analysis aimed to determine the primary factor influencing the distribution of PA, enabling the identification of reasons for regional differences in PA duration and the formulation of targeted PA policies for each area. Secondly, we investigated the correlation between chronic diseases and PA to explore the amount of PA per week associated with a lower risk of developing chronic diseases. This information will play a crucial role in guiding the development of PA programs for older adults, focusing on five specific chronic diseases: heart disease, cerebrovascular disease, lumbar and cervical spondylosis, arthritis or rheumatism, and respiratory diseases. Lastly, we examined the impact of personalized factors on PA among older adults, which is essential for developing tailored measures and guidance for promoting PA at an individual level.

## Methods

### Study design and participants

This study utilized a cross-sectional design and analyzed data from the CLASS. The CLASS project is a national, large-scale social survey conducted by the National Survey Research Center at Renmin University of China (NSRC). The survey covered 28 provinces, 134 districts, and 462 villages across China, excluding Hong Kong, Taiwan, Macao, Hainan, Xinjiang, and Tibet. The participants in this study were 11,396 Chinese citizens aged 59 years and above who were surveyed using a questionnaire administered by CLASS. The survey was conducted in 2020 based on data from the first three rounds of the survey, which took place from 2011 to 2020. The study protocol was approved by the Ethics Committee at the Capital University of Physical Education and Sport (ChiCTR-IOR-ChiCTR2200063177).

### Survey procedures

Data collection for the CLASS project was carried out using the China Social Survey Network (CSSN). The survey employed a stratified, multi-stage, probability sampling method. County-level regions, including counties, county-level cities, and districts, were selected as primary sampling units (PSUs). Villages and neighborhood committees were chosen as secondary sampling units (SSUs). Sample households were then selected using a random sampling method within each village/neighborhood committee, with one elderly person interviewed in each household. Data collection involved interviews and questionnaires. To ensure data quality, on-site supervision and call-backs were employed as part of the quality control measures implemented by CLASS.

### Assessment of physical activity level

The participants’ PA levels were assessed using self-report structured questionnaires based on a 7-day recall method. The questionnaires captured information on the intensity, frequency, and motivations for participating in PA. To aid participants’ understanding, examples of different types of physical activities were provided. For instance, moderate-intensity physical activities (MPA) were exemplified by activities such as light-weightlifting, cycling at a moderate pace, or playing tennis or badminton. The survey included questions on the usual frequency (number of days per week) and total duration (in minutes) of vigorous-intensity physical activity (VPA), MPA, light-intensity physical activity (LPA), and sedentary time. Physical activities lasting at least 10 min were considered in the calculations. VPA was defined as activities requiring significant effort, causing shortness of breath, faster heart rate, increased sweating, such as heavy-weight lifting, digging, aerobics, and high-speed cycling. MPA referred to exercise causing mild shortness of breath, faster heart rate, and slight sweating. LPA encompassed activities with minimal impact on breathing and heart rate, such as slow or leisurely-paced walks. Sedentary activity accounted for all sitting time. The energy expenditure of physical activities was calculated using metabolic equivalent (MET) values based on previous studies [40], and the intensity of each activity was categorized according to the World Health Organization (WHO) guidelines [41]. The equations used for calculation are listed below [42].

$$\begin{array}{l}\mathrm{LPA\, MET}-{\text{minutes}}/\mathrm{week }= 3.3*\mathrm{total\, walking\, time\, in\, a\, week}\\ \mathrm{MPA\, MET}-{\text{minutes}}/\mathrm{week }= 4.0*\mathrm{total\, MPA\, activity\, minutes\, time\, in\, a\, week}\\ \begin{array}{l}\mathrm{VPA\, MET}-{\text{minutes}}/\mathrm{week }= 8.0*\mathrm{total\, VPA\, activity\, minutes\, time\, in\, a\, week}\\ \mathrm{Total\, PA\, MET}-{\text{minutes}}/\mathrm{week }=\mathrm{ sum\, of\, LPA }+\mathrm{MPA }+\mathrm{ VPA\, MET}-{\text{minutes}}/\mathrm{week\, scores}\end{array}\end{array}$$

Inactive was identified if PA amount in a week failed to meet at least one of the following levels according to International Physical Activity Questionnaire (IPAQ) group, while the rest were classified as active.

- 3 or more days of VPA activity of at least 20 min per day

OR

b) 5 or more days of MPA activity and/or LPA of at least 30 min per day

OR

c) 5 or more days of any combination of LPA, MPA or VPA activities achieving a minimum total PA of at least 600 MET-minutes/week

### Statistical analysis

In this study, the durations of PA for all participants were converted to minutes per week and expressed as means for various analysis purposes. To facilitate further analysis, geographical, economic, and urbanization factors were discretized. Provinces not intersected by the Heihe-Tengchong Line are categorized as ‘west areas’ if they lie to the left of the line, and as ‘east areas’ if they lie to the right of the line. Provinces intersected by the Heihe-Tengchong Line are classified based on the surveyed cities within each province. If the surveyed city is situated to the left of the Heihe-Tengchong Line, the province is categorized as ‘west areas’, if it is to the right, it is categorized as ‘east areas’. For a more detailed breakdown, please refer to Table S1 in the Supplementary Appendix. The specific classification criteria are detailed below:

- Geography: The Heihe-Tengchong line [43] was adopted as the geographic divider, dividing China into east areas and west areas based on contrasting population densities. The east areas included Heilongjiang, Jilin, Liaoning, Beijing, Tianjin, Hebei, Shanxi, Sichuan, Yunnan, Shandong, Henan, Jiangsu, Shanghai, Zhejiang, Hunan, Hubei, Chongqing, Jiangxi, Guizhou, Guangdong, Guangxi, and Fujian. The west areas included Inner Mongolia, Ningxia, Gansu, Shaanxi, and Qinghai.
- Economy status: Gross domestic production (GDP) per capita in 2020 was used to evaluate the economic divider. High GDP areas included Beijing, Shanghai, Jiangsu, Fujian, Zhejiang, Guangdong, Tianjin, Chongqing, Hubei, and Shandong. Low GDP areas included Inner Mongolia, Shaanxi, Anhui, Hunan, Sichuan, Liaoning, Henan, Ningxia, Jiangxi, Yunnan, Qinghai, Guizhou, Hebei, Shanxi, Jilin, Guangxi, Heilongjiang, and Gansu.
- Urbanization: Participants were distinguished based on their living area, either urban or rural, as provided by the survey.

Descriptive data is presented using measures such as mean, standard deviation, numerical values, and percentages. Before proceeding with further analysis, we assessed the normality of each variable by conducting the Kolmogorov-Smirnov normality test to determine if the data follows a normal distribution. In the investigation of PA levels, we applied the Wilcoxon rank sum test to evaluate the statistical significance between different groups. We employed Bayesian networks to analyze the impact of socio-economic factors on PA. The construction and learning of the model were performed using Python 3.8.10, with the bnlearn package (version 0.8.0) utilized. The allocation of variables was documented in Table S2 within the supplementary appendix. During the analysis process, we transformed the continuous variable representing the duration of PA into a categorical variable by calculating quartiles for different intensity levels and assigning corresponding values. For the duration of PA, a value of 0 was retained as 0, while other durations were assigned values of 1, 2, 3, and 4 based on the quartile calculations, respectively. Participants with a duration of 0 for MPA and MVPA were excluded when calculating the quartiles. To ensure that the PA node served as the end node of the network, we utilized the ‘black_list’ parameter to prevent the influence of PA on the other four factors. We employed a hill-climbing algorithm for learning and applied the Bayesian method to calculate the conditional probability between each node, and the nodes used to construct the Bayesian network structure included economic status, geography, living areas (urban and rural), HDI, and the duration of PA. In our study investigating the correlation between chronic diseases and PA, we employed the chi-square test to examine the relationship between various chronic diseases and PA. The chronic diseases under investigation included heart disease, cerebrovascular disease, lumbar and cervical spondylosis, arthritis or rheumatism, and respiratory diseases. Furthermore, we utilized the chi-square test to analyze the impact of personalized factors on PA participation. Participants provided responses to the questionnaire, specifically addressing factors influencing their engagement in PA. Within the questionnaire, when participants selected a particular factor, it was coded as 1, and if not selected, it was coded as 0. All statistical tests were two-tailed, and p-value < 0.05 was considered statistically significant. The statistical analyses were conducted using Python version 3.8.10.

## Results

We compiled a comprehensive dataset comprising 11,396 older adults aged over 59 years from 28 provinces in China, utilizing data from the CLASS. The dataset encompasses a range of sociodemographic characteristics, including information on Body Mass Index (BMI), the number of chronic diseases, and other relevant participant characteristics. These details are presented in Table 1, providing a succinct overview of the dataset. Among 11,396 participants, the mean (SD) age was 71.6 (6.6) years (range, 59–100 years), 5649 (49.6%) were women, and 5747 (50.4%) were men. 7218(63.3%) individuals were normal weight (18.5 kg/m^2^ ≤ BMI ≤ 23.9 kg/m^2^), 3165 (27.8%) were overweight (23.9 kg/m^2^ < BMI ≤ 27.9 kg/m^2^), 596 (5.2%) were thin (BMI < 18.5 kg/m^2^) and 417 (3.7%) were obese (BMI > 27.9 kg/m^2^). The population of smokers was 3174 (27.9%). Overall, participants in 28 provinces spent a mean (SD) of 737.06 (643.66) minutes per week in LPA, 39.45 (134.24) minutes in MPA and 48.17 (161.03) minutes in MVPA. The mean value of sedentary behavior over the week was 1425 (758.06) minutes. The WHO recommended in its 2020 guidelines on PA and sedentary behavior that older adults should participate in at least 150–300 min of moderate-intensity aerobic PA or 75–150 min of vigorous-intensity aerobic PA or an equivalent combination throughout per week. As a result, our study showed the PA level of China’s older adults is less than a third of the level recommended by the WHO and the prevalence of PI among the older adults over 59 years in China is 28.82% during 2020.

**Table 1:** Participant demographic characteristics

**Characteristic**	**Sample**
Total	11,396 (100%)
Sex	
Male	5747 (50.4%)
Female	5649 (49.6%)
Age	
59–69	5032 (44.16%)
70–79	4744 (41.63%)
80–89	1529 (13.42%)
90–98	91 (0.79%)
BMI	
Thin	596 (5.2%)
Normal weight	7218 (63.3%)
Overweight	3165 (27.8%)
Obese	417 (3.7%)
Number of chronic diseases	
No chronic disease	2999 (26.3%)
One chronic disease	3152 (27.7%)
Two chronic diseases	2670 (23.4%)
Three chronic diseases	1568 (13.8%)
Four or more chronic diseases	1007 (8.8%)
Smoking	
Yes	3174 (27.9%)
No	8222 (72.1%)
Education	
Illiterate	2670 (23.5%)
Private school/ literacy class	482 (4.2%)
Junior school	4191 (36.8%)
Junior high school	2819 (24.7%)
> High school graduate	1234 (10.8%)
Type of area of residence	
The central area of the city/county	3928 (34.5%)
The edge of the city/county	1162 (10.2%)
Urban-rural junction of city/county	695 (6.1%)
Towns outside the city/county area	487 (4.3%)
Countryside	5124 (44.9%)
Average of PA	
Sedentary Behavior, mean (SD), h	1425 (758.06)
LPA, mean (SD), min	737.06 (643.66)
MPA, mean (SD), min	39.45 (134.24)
MVPA, mean (SD), min	48.17 (161.03)

**Fig. 1:**
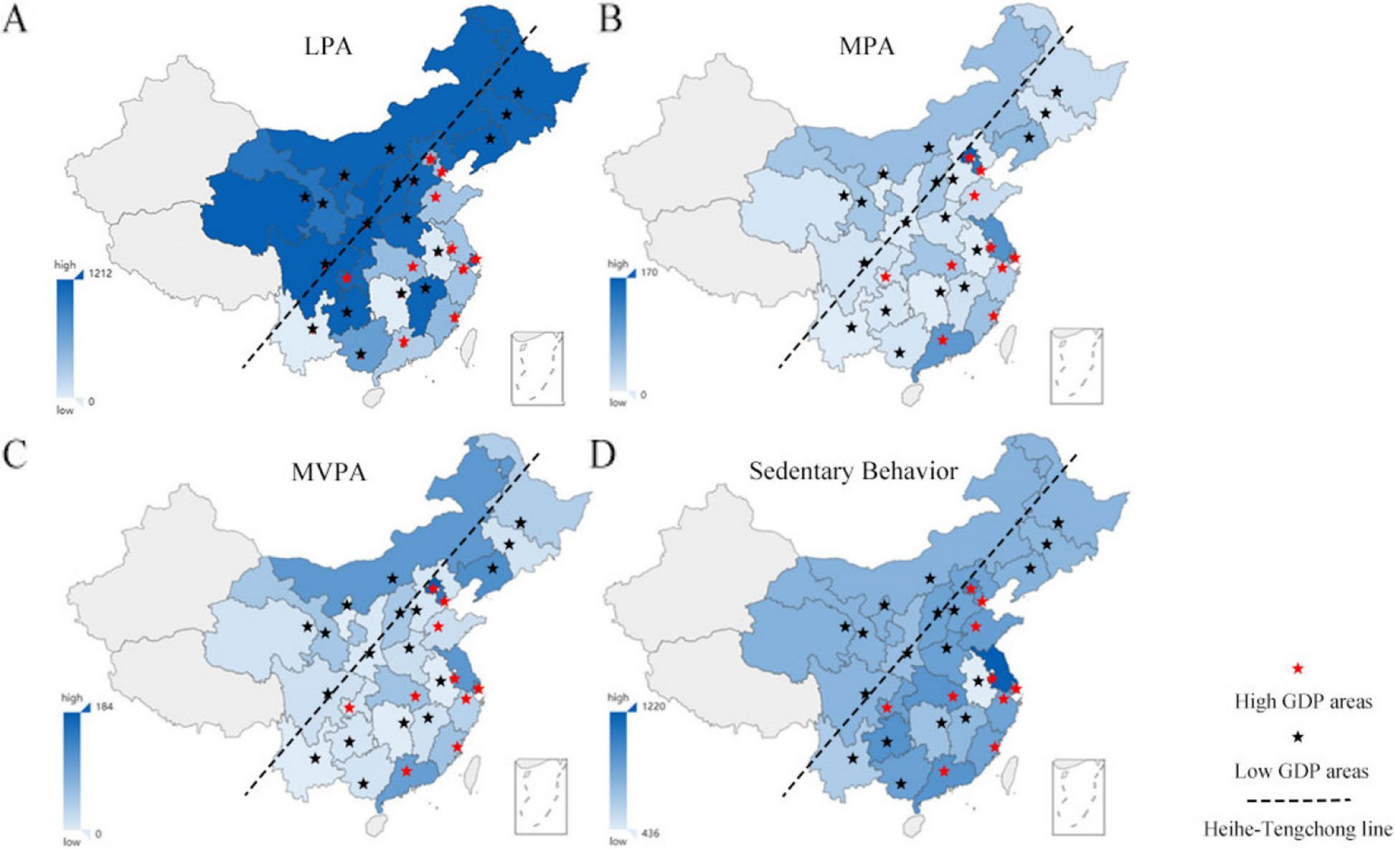
The distribution of different types of physical activity by average length of time. The unit for four figures is minutes/wk. The maps used in this article are from Google Maps. The grey areas are the Xinjiang, Tibet, Hainan, Hongkong, Macaw, Taiwan which are not included in our survey. The star symbol indicates the location of the provincial capitals

**Fig. 2:**
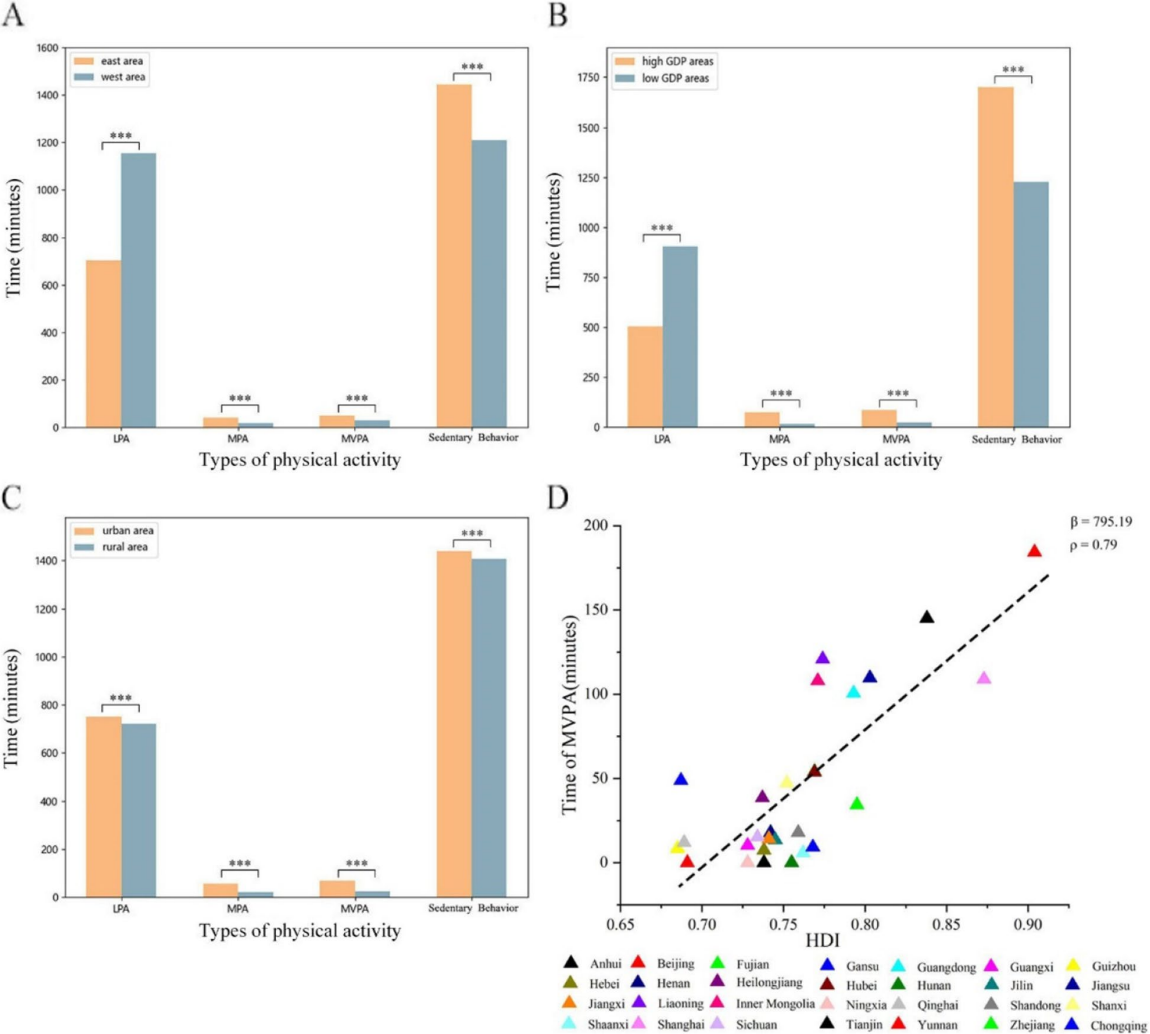
The relationship between physical activity and socio-economic factors. The relationship between physical activity by average length of time and geography (**A**), economic status (**B**), living areas (urban and rural) (**C**), HDI (**D**). ***: *p* < 10^–3^, **: 10^–3^ ≤ *p* ≤ 10^–2^, *:10^–2^ ≤ *p* ≤ 10^–1^

We constructed a map of PA of different intensities among the older adults in China, and analyzed the significant differences in the specific duration of PA of different intensities based on geographic and economic factors. The map of PA is shown in Fig. 1. The distribution of PA time is illustrated in the Fig. 2A, B, and C. There are significant differences in the specific duration of PA divided by Heihe-Tengchong line, economy status and living areas (urban and rural). The older adults in the east areas have a longer time of MPA (41.16:18.74[east area: west area, unit: min per week.]) (*p* = 4.69×10^-7^, Wilcoxon rank sum test), MVPA (49.85:27.89) (*p* = 2.75×10^-6^) and sedentary behavior (1443:1208.4) (*p* = 1.61×10^-13^) and a shorter time of LPA (702.54:1154.58) (*p* = 6.12×10^-31^) than the older adults in west areas. The older adults in the high GDP areas have a shorter length of time on LPA (503.94:903.08[high GDP areas: low GDP areas, unit: min per week.]) (*p* = 0.000) and longer time of MPA (75.35:13.90) (*p* = 2.27×10^-150^), MVPA (83.88:22.77) (*p* = 3.03×10^-137^), and sedentary behavior (1702.20:1227.6) (*p* = 1.32×10^-182^) than the older adults in low GDP areas. The older adults live in urban areas spent more time on MPA (55.41: 20.36[urban area: rural area, unit: min per week.]) (*p* = 3.58×10^-43^), MVPA (67.27:24.8) (*p* = 6.13×10^-45^), LPA (750.36:720.94) (*p* = 0.015) and sedentary behavior (1438.8:1408.2) (*p* = 0.03) than the older adults live in rural areas. To further examine the data, we introduced the HDI as an additional variable. The findings demonstrated that individuals residing in areas with higher HDI scores tend to engage in longer durations of MPA, MVPA, and sedentary behavior, while spending less time on LPA. These distribution patterns and trends are visually depicted in Fig. 2D. In summary, the specific duration of PA exhibits variations across geographical, economic status, and living area factors. Older adults residing in economically developed regions demonstrate higher engagement in MPA and MVPA activities.

**Fig. 3:**
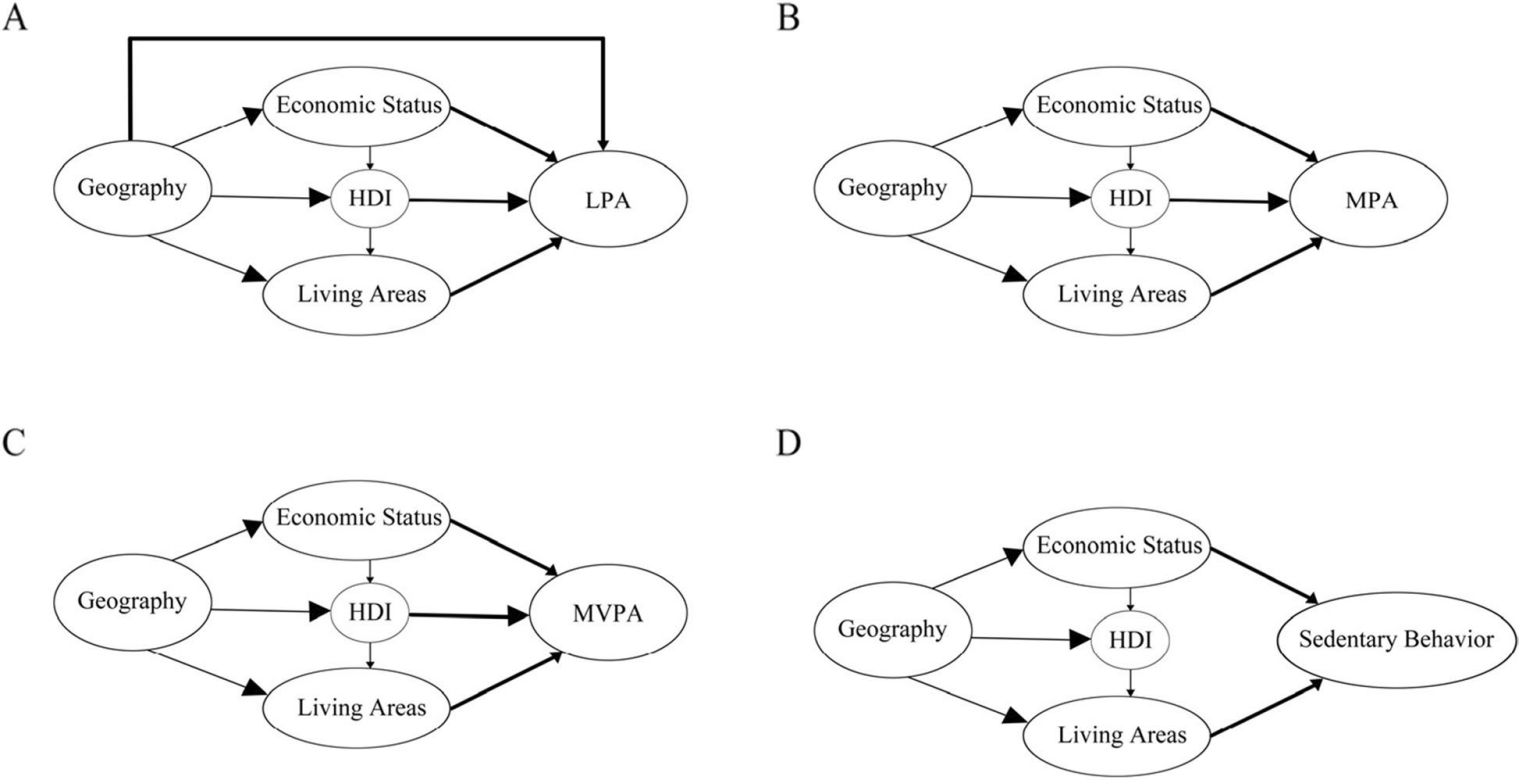
Bayesian network structure learning results for the impact of different factors on physical activity

We conducted a Bayesian network analysis to investigate the socio-economic factors that directly influence PA. We employed bold black arrows to symbolize the socio-economic factors that have a direct impact on LPA, MPA, MVPA, and sedentary behavior (Fig. 3). The results consistently revealed that economic status and living areas (urban and rural) emerged as direct influencing factors for PA across all intensity types. To provide a more detailed understanding of the relationships between various factors, we conducted an analysis of conditional probabilities for each node. The results of this analysis can be found in Tables S3.1–6.5 in the supplementary appendix. For instance, we observed that living areas (urban and rural) and economic status are two key factors that directly influence sedentary behavior. When both living areas (urban and rural) and economic status are categorized as “1”, the probability of an individual’s sedentary behavior duration exceeding 28.1 h per week is 32.17%. However, when both are categorized as “0”, this probability drops to 11.01%. This finding suggests that older adults residing in developed areas are more likely to engage in sedentary behavior compared to their counterparts in underdeveloped areas.

**Fig. 4:**
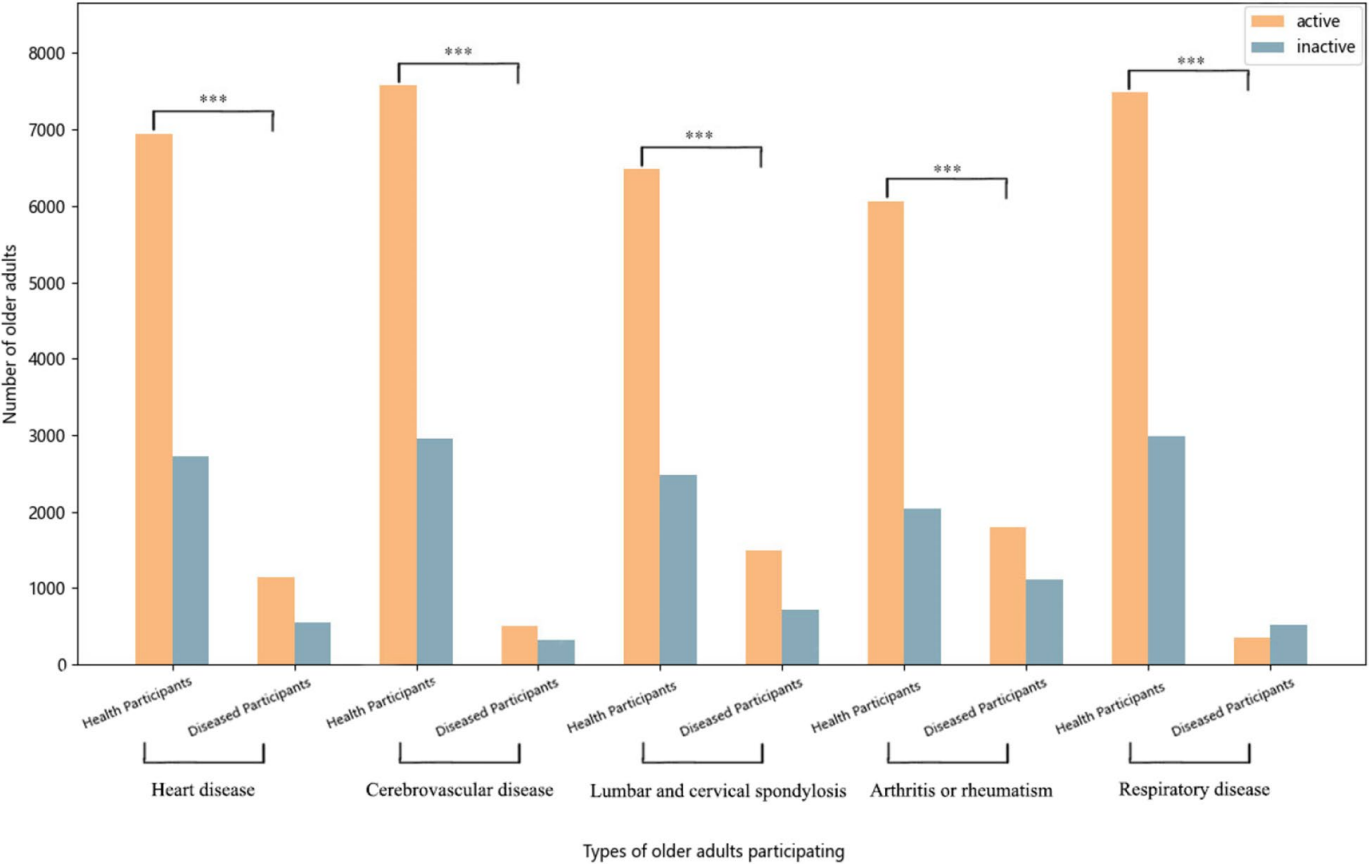
Association between illness and physical activity. ***: *p* < 10^–3^, **: 10^–3^ ≤ *p* ≤ 10^–2^, *: 10^–2^ ≤ *p* ≤ 10^–1^

To examine the correlation between PA and chronic diseases, as well as determine the amount of PA at different intensities associated with a lower risk of chronic diseases, we performed statistical analysis on the participants’ chronic disease status. The chronic diseases considered in this study include heart disease, cerebrovascular disease, lumbar and cervical spondylosis, arthritis or rheumatism, and respiratory diseases. Participants were categorized into active and inactive groups based on the criteria outlined in the “Physical Activity Level Assessment” section of the Methods. This categorization applied to both participants with and without the mentioned chronic diseases. Subsequently, we performed a chi-square test to examine the correlation between levels of PA and the presence of chronic diseases, as depicted in Fig. 4. The number and proportion of active participants diagnosed with heart disease, cerebrovascular disease, lumbar and cervical spondylosis, arthritis or rheumatism and respiratory diseases were 1136 (67.50%), 506 (61.11%), 1491 (67.71%), 1794 (61.59%) and 358 (41.10%), respectively. The number and proportion of active group in the population without the above five chronic diseases are 6936 (71.77%) (*p* = 0.0004), 7583 (71.99%) (*p* = 3.75*10^-11^), 6479 (72.29%) (*p* = 2.23*10^-5^), 6055 (74.76%) (*p* = 2.87*10^-41^), and 7490 (71.46%) (*p* = 3.56*10^-77^), respectively. The result shows that among the five chronic diseases, the proportion of active participants in the healthy participants is significantly higher than the proportion in the sick participants. This can probably indicate that regular PA has a positive impact on the health of older adults. When the amount of PA within a week reaches a certain standard, it has a positive effect on reducing the risk of chronic diseases.

**Fig. 5:**
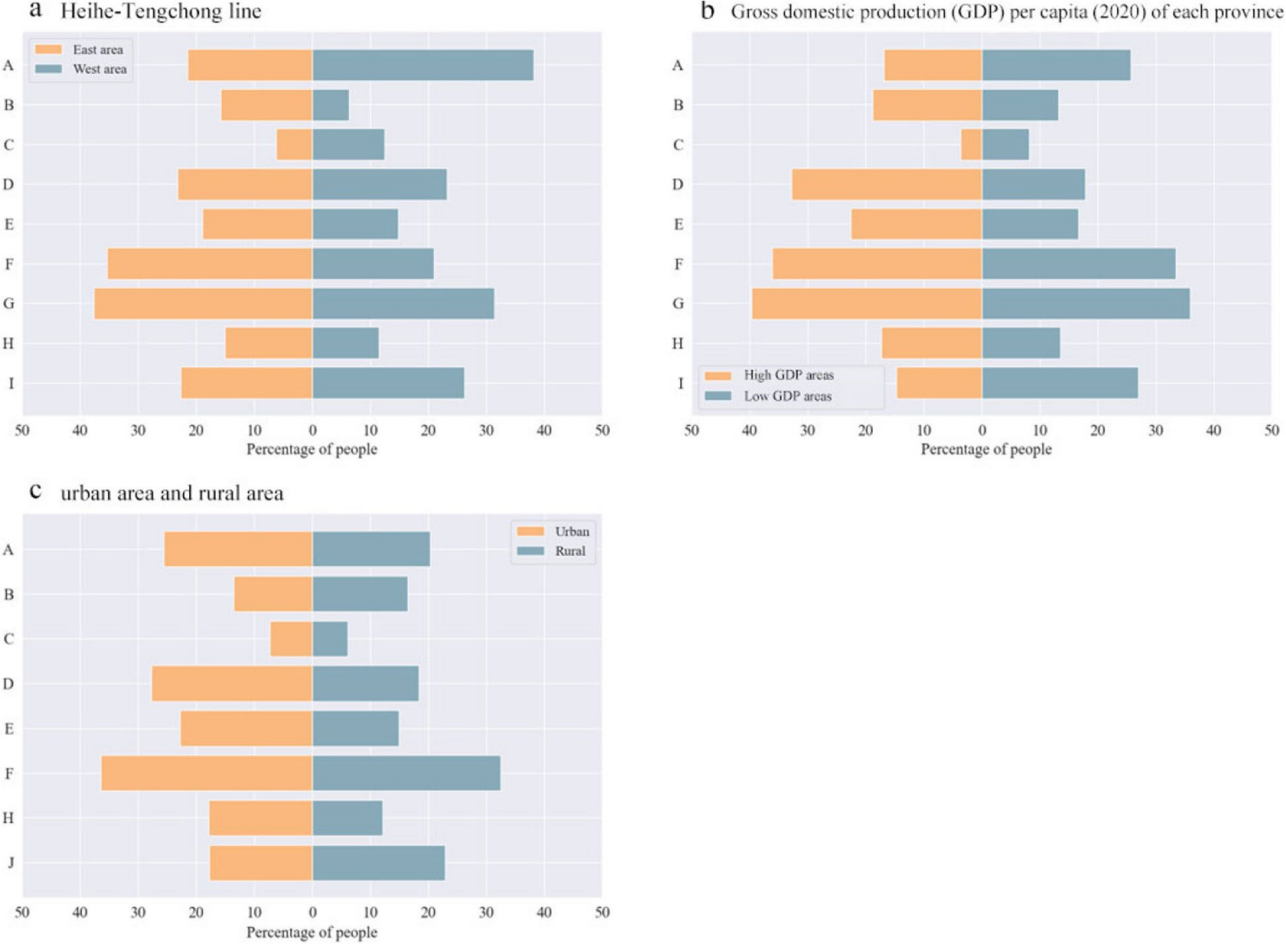
Reasons for not attending physical activity. **A** Laziness. **B** Insufficient knowledge for physical activity and fitness knowledge. **C** Babysitting occupies physical activity time. **D** No support from sons or daughters. **E** No support from spouse. **F** Instruction needed. **G** Need host of physical activity event. **H** Activity content not applicable. **I** Lack of venue facilities. **J** No need because of extensive labor

We examined the personalized factors that impact PA among older adults by employing a chi-square test in the specified regions, as elaborated in the Statistical Analysis section of our Methods. China was divided into east areas and west areas, high GDP areas and low GDP areas, as well as rural and urban areas. The personalized factors that significantly influenced older adults’ PA are presented in Fig. 5. The *p*-values associated with these personalized factors are listed in Table 2. The results revealed that among factors such as insufficient knowledge about physical activity and fitness knowledge, inapplicable activity content, the need for a host for physical activity events, and the need for instruction, the proportion of older adults in the east areas and high GDP areas was significantly higher than that in the west areas and low GDP areas. However, the factors of laziness and lack of venue facilities showed the opposite trend. Regarding family factors, the proportion of older adults who engaged in less PA due to a lack of support from their sons, daughters, and spouse was significantly higher in the east areas and high GDP areas. Conversely, the factor of babysitting occupying physical activity time showed the opposite result. Furthermore, in rural areas, a significantly higher proportion of individuals reported not engaging in PA due to the lack of need resulting from extensive labor, as compared to urban areas. In conclusion, personalized factors such as laziness, lack of venue facilities, inadequate social guidance, and lack of family support are modifiable factors that influence PA among older adults. The impact of these factors is associated with geographical, economic, and living area differences. These findings highlight the importance of addressing these factors to promote PA among older adults.

**Table 2:** *P* value for testing the difference of the factors in the divided areas

**Personalized factors**	***P* value of east areas vs west areas**	**χ**^**2**^	***P* value of high GDP areas vs low GDP areas**	**χ**^**2**^	***P* value of urban area vs rural area**	**χ**^**2**^
Laziness	1.26*10^–17^	73.06	8.54*10^–16^	64.74	3.81*10^–7^	25.79
Insufficient knowledge for physical activity and fitness knowledge	9.36*10^–9^	32.97	1.87*10^–9^	36.10	0.001	10.54
Babysitting occupies physical activity time	7.97*10^–8^	28.81	4.27*10–^12^	48.00	0.046	3.98
No support from sons or daughters	0.004	8.40	1.79*10^–42^	186.56	1.40*10 ^−19^	81. 95
No support from spouse	0.023	5.17	6.06*10^–9^	33.82	1.50*10^–16^	68.17
Instruction needed	4.06*10^–11^	43.59	0.034	4.51	0.001	11.92
Need host of physical activity event	0.006	7.65	0.003	8.93	0.465	0.54
Activity content not applicable	0.027	4.87	0.0001	15.81	5.52*10^–11^	42.98
Lack of venue facilities	0.063	3.45	4.79*10^–29^	125.12	0.923	0.01
No need because of extensive labor	8.43*10^–12^	46.66	0.111	2.55	1.88*10^–7^	27.15

## Discussion

The aging process in China is advancing at a rapid pace, marked by a substantial proportion of elderly individuals, and their distribution covers an extensive geographic range. Unlike many other countries worldwide, China’s elderly population spans a broader spectrum of longitudes and latitudes. This characteristic adds complexity and diversity to the challenges associated with aging in China, while also offering a wealth of data and case studies for research on the aging population. Through an analysis of China’s elderly population, we can delve into factors that influence aging, stage-specific characteristics, regional disparities, and other related issues. This research provides valuable insights and inspiration not only for other countries and regions but also for addressing the unique complexities of aging in China. Existing studies have primarily focused on specific regions within China [44–46]. Moreover, there is a notable absence of nationwide, large-sample studies during the COVID-19 epidemic. This study aims to investigate the specific amount of PA among older adults in China in 2020 and analyze the characteristics of regional distribution. It examines the influence of socioeconomic factors such as GDP, urban-rural and geographic environments, as well as personalized factors on PA. Additionally, the study explores the relationship between PA and the reduced risk of chronic diseases among older adults. To our knowledge, this study represents the pioneering effort to investigate the socio-economic patterns of PA among older adults in China amid the backdrop of the COVID-19 pandemic. The primary discoveries of this study can be summarized as follows:

1. The PA level of older adults in China is lower compared to the global average. The prevalence of PI among older adults in China is 28.82%.
2. Geographical, economic, and urban-rural differences exist in the distribution of PA among older adults in China. Our findings consistently demonstrate that economic status and living areas (urban and rural) have a significant direct influence on PA across all intensity levels. Older adults residing in more economically developed areas engage in higher levels of MPA and MVPA.
3. Active older adults have a significantly lower risk of chronic diseases compared to inactive older adults.
4. Personalized factors such as laziness, lack of social guidance, and insufficient family support play a significant role in promoting active participation in PA among older adults. These factors are relatively easier to address and modify.

The PA level of older adults in China is lower than the recommended level by the WHO and some developed countries, although the prevalence of PI has decreased compared to the survey results in China 10 years ago. The CHARLS project previously reported a prevalence of PI among Chinese adults aged 45 and above was 44.06% between 2011 and 2012 [37]. Our results showed that the prevalence of PI has decreased to 28.82% among older adults aged 59 and above in China in 2020. A few international studies that using questionnaires or the accelerator data to measure PA level. Jason A Bennie et al. [47] found in 32 EU countries, their sedentary time of 36.52 h/week for the elders aged 65 years and older which is 54% more than the time in our study. A Spanish study [48] reported that the mean values of VPA is 38.3 min/week, MPA is 390.1 min/week, and MVPA is 428.4 min/week for older adults aged 65 to 75 years. Their result is higher than both the WHO recommendations, and the mean value of Chinese older adults. One study [49] using accelerometer investigate the MPA and MVPA time length for different age groups. The result of age group over 60 is 71.4 min/week and 72.4 min/week for MPA and MVPA respectively, which didn’t meet the WHO recommendations, while still more than the counterpart in our study. This phenomenon may be associate with the exercise awareness of Chinese is still remain lower than the most countries in the world. The exercise awareness is needed to be raised up.

In this study, we found that GDP and HDI are positively correlated with MVP, MVPA and sedentary behavior, and negatively correlated with LPA. People who live in the high GDP or HDI areas tend to adopt a habit that has a low LPA level, while they attend more MPA, MVPA and sedentary behavior than people who live in lower GDP areas. Our findings are in line with some of the previous study [50–52], in which stress that GDP and HDI are associated with the MVPA or leisure time physical activity (LTPA) level. However, there are also results that are inconsistent with this finding. Bosdriesz et al. [53] discovered an inverse tendency of VPA and GDP. A 76 countries study is also inconsistent with our study: HDI spurs inactivity (< 2.5 h MPA per week).

We also found that the people live in urban and the people in rural area have diverse PA habits. Urban citizens spent more time on LPA, MPA, MVPA and sedentary behavior. This finding has similarities with previous studies. A European study [51] focusing on LTPA indicates that the LTPA participation was positively associated with GDP, affluence, and urban area size. One study [53] covering 38 countries found that economic growth influenced MVPA and sedentary time among Europeans and found a positive association between GDP and sedentary time for people over 65 years old. Older people living in urban areas stimulated the increase in sedentary time compared to rural areas influenced by their living environment, community security, transportation status, and level of social connections. Whereas, rural older adults increase their overall PA levels due to farming and gardening activities. One previous Chinese study [50] showed a weak positive correlation between GDP per capita and PA in China, i.e., people in economically developed areas engage in more PA than those in less developed areas.

Our study suggested that the level of PA is highly correlated with geography, similar results can be found in previous researches [47, 54–56], but few researches have been conducted specifically on older age groups. Further corroborating evidence comes from United States and Iran and is consistent with our results [47, 54, 57], they found that the areas with better living conditions had less LTPA and more VPA, while the areas with worse living conditions had more LTPA and less VPA. This may be due to local differences in climate, topography or ecological resources. However, northwest European countries generally report higher sedentary time than southeast European countries, possibly because more people in Northwest Europe work in white-collar or office-based occupations that require more time sitting. In contrast to our findings [56], less sedentary time was found in coastal areas and more sedentary time was found in inland areas, as coastal areas may be a preferred living environment for people who value PA. This study found that the distribution of the specific duration of PA was affected by geographical factors, economic status factors and living areas factors. Through the analysis of Bayesian networks, we consistently found that economic status and living areas (urban and rural) are the primary direct influencing factors of PA among all the factors considered.

Regular PA has a positive impact on the health of the older adults, and achieving a certain duration of PA at different intensities is associated with the lower chronic diseases risk. In our study, healthy participants and chronic disease patients were divided into active and inactive respectively, according to the IPAQ scoring standard [42]. We found that among the five chronic diseases, the proportion of active in the healthy participants is significantly higher than the proportion in the sick participants. This suggests that regular PA has a positive impact on the health of older adults. This phenomenon has been reported by other studies previously. BARKER et al. [58] found that participants without chronic disease performed an average of 61 min more MPA per week than those with chronic disease. Darren E.R. Warburton and colleagues [59] found that regular PA is beneficial for health. Regular PA plays a significant role in the primary and secondary prevention of various chronic diseases and is associated with a reduced risk of premature death. ASHE et al. and LESKINEN et al. [60, 61] observed that people who were active lived longer and had a lower risk of death compared to those who did not exercise, which is similar to our study.

Based on the findings regarding the low compliance rate of active participation in PA among older adults in China and the personalized factors affecting their PA, we propose several recommendations:

1. Addressing Geographic and Economic Disparities: The study has unveiled disparities in PA levels and the influencing factors among older adults, based on geographic and economic considerations. Older adults residing in economically more developed regions showed a greater inclination towards engaging in MPA and MVPA. Simultaneously, older adults actively participating in PA were associated with a lower risk of chronic diseases. To address these disparities, it is recommended that the government formulate region-specific PA policies. These policies should be tailored to the economic conditions of different regions and the distinctions between urban and rural areas, with the aim of better catering to the needs of older adults across diverse geographic contexts. Notably, in economically developed areas, older adults exhibit a higher motivation to engage in MPA and MVPA. However, in less developed areas, there is a need for efforts to stimulate economic growth and implement relevant policies and systems. Furthermore, our study has revealed that the prevalence of chronic diseases in some provinces and cities in China surpasses the national average, even in regions with strong economic development (Supplementary Appendix Table S7). This underscores the urgency of increasing the PA participation rate among older adults in these areas. We have calculated the prevalence of five chronic diseases in different provinces (refer to Table S7 of the supplementary appendix). For instance, the results indicate that the diagnosis rates of heart disease in Beijing and Shanghai stand at 21.08% and 24.82%, respectively, which exceed the national average of 14.77%. While older adults in these economically developed areas are more inclined to engage in MPA and MVPA, they also face relatively high rates of heart disease diagnosis. For economically developed regions, considering the key personalized factors identified in this study that impact PA, we propose the following policy recommendations to provide support: 1). Enhance the provision and construction of PA facilities. 2). Increase public awareness and education regarding PA through science dissemination and guidance.
2. Implementation of a PA monitoring network for older adults: Our findings showed that PA levels of older adults in China are lower than the global average. We propose to establish a system that regularly measures and evaluates the PA levels of older adults over the course of a week to better understand and promote the current situation and development trend of PA. This network can help assess compliance rates with PA guidelines, contribute to better prevention of chronic diseases and help governments adjust policies in a timely manner.
3. Enhancing the public service system for older adults: Our findings have revealed that, among personalized factors, a lack of physical activity and fitness knowledge, the need for instruction, and irrelevant activity content are key factors influencing the participation of older adults in PA. This situation is largely attributed to the shortcomings of the current public service system. Government agencies should focus on enhancing and modernizing the existing public service infrastructure. Specific strategies may involve increasing the number of trained social sports instructors, providing PA facilities tailored to the needs of older adults, and enhancing the accessibility and availability of community resources.
4. Increasing Public Awareness of the Benefits of PA: Our research has highlighted that several personalized factors, such as a lack of motivation, responsibilities like babysitting that consume PA time, and insufficient support from family members, notably impact the participation of older adults in PA. It is crucial to enhance awareness among older adults regarding the numerous advantages of regularly engaging in PA. Communities can play a pivotal role in achieving this goal. Local healthcare providers can host regular health lectures to disseminate relevant knowledge to older adults, their family members, and spouses, while offering accessible information and guidance on PA. Furthermore, the use of informative posters and the distribution of promotional materials can be effective in raising public awareness.

In conclusion, this study, which makes a pioneering effort to explore the relationship between PA and socio-economic status, holds great potential for strengthening the connection between local regions and promoting activity and health. Furthermore, the findings can inform policy making at the local, provincial, and national levels.

### Limitations and strengths

This study has a few limitations that should be acknowledged. Firstly, the study relied on the IPAQ to assess PA levels among older adults, which may introduce subjectivity and potential recall bias. The use of objective measures, such as accelerometers or wearable devices, could have provided more accurate and reliable data on the actual PA levels of the participants. Secondly, the study employed a complete case analysis approach to handle missing data. While this is a commonly used method, it has the potential to introduce bias if the missing data are not missing completely at random. Future studies could consider using more robust techniques, such as multiple imputation, to address missing data and provide more robust results. Thirdly, this study lacks a comparative analysis with other regions and time periods. However, due to the unavailability of reliable and consistent data on the PA of older adults in other regions and time periods, this study was unable to conduct such comparative analysis. Therefore, the generalizability of the findings may be limited to the Chinese context and the situation in 2020. Future research will include the collection and comparison of data from different regions and time periods, with the aim of exploring potential variations and similarities in PA among elderly individuals in various contexts.

On the other hand, the study has notable strengths. It is a trailblazing study that quantitatively analyzes the specific duration of PA based on large-scale surveys conducted in multiple provinces in China. This approach allows for a more comprehensive understanding of the time spent on different intensities of PA among older adults. Furthermore, the study explores the personalized factors influencing PA and examines their correlations with health outcomes, which provides valuable insights for designing targeted interventions and promoting healthy aging.

In summary, while this study has limitations, such as the use of self-reported measures and the approach to handling missing data, its strengths lie in the comprehensive analysis of PA duration, personalized factors, and their association with health outcomes among older adults in China. Future research can build upon these findings by incorporating objective measures and employing more robust methods for data analysis.

## Conclusions

Our study sheds light on the PA levels of older adults in China, revealing that they fall below the recommended levels set by the WHO. Through a comprehensive quantitative analysis, we examined the influence of socio-economic differences on PA among older adults, providing valuable insights into this population segment in a developing country.

One significant finding is the relatively high prevalence of PI among older adults in China, although there has been a decrease compared to previous years. We observed variations in the duration of PA at different intensities based on economic status, geographical region, and living areas (urban and rural). The Bayesian network analysis revealed that economic status and living areas (urban and rural) have a direct impact on PA. Specifically, older adults residing in economically developed areas exhibit higher levels of engagement in MPA and MVPA. These findings highlight the influence of economic factors and living environment on the activity levels of older adults. Furthermore, our study demonstrates a clear association between active lifestyles and a lower risk of developing chronic diseases among older adults. Personalized factors, including laziness, lack of social guidance, and inadequate family support, emerged as significant influencers of PA in this population. These findings provide a crucial foundation for national and local governments to develop targeted health promotion action plans for older adults, bridging the existing gap in understanding the PA status of older adults in developing countries.

The implications of our study extend to both primary and secondary prevention of chronic diseases among older adults. It underscores the importance of interventions that address personalized factors and encourage active participation in physical activities. Moving forward, we plan to conduct further investigations, including transforming our study into a prospective cohort study to track the longitudinal changes in PA levels among Chinese older adults. By addressing the limitations identified in this research and expanding our scope, we aim to contribute to a deeper understanding of the PA patterns among older adults in China and provide valuable insights for developing effective strategies to promote healthy aging in this population.

### Supplementary Information

**Additional file 1: Table S1.** The basis for dividing the east areas and west areas. **Table S2.** Variables and their assignments in the Bayesian Network model. **Table S3.1.** The conditional probability of the Geography node in Fig. 3A. **Table S3.2.** The conditional probability of the Economy status node in Fig. 3A. **Table S3.3.** The conditional probability of the HDI node in Fig. 3A. **Table S3.4.** The conditional probability of the Living Areas node in Fig. 3A. **Table S3.5.** The conditional probability of the LPA node in Fig. 3A. **Table S4.1.** The conditional probability of the Geography node in Fig. 3B. **Table S4.2.** The conditional probability of the Economy status node in Fig. 3B. **Table S4.3.** The conditional probability of the HDI node in Fig. 3B. **Table S4.4.** The conditional probability of the Living Areas node in Fig. 3B. **Table S4.5.** The conditional probability of the MPA node in Fig. 3B. **Table S5.1.** The conditional probability of the Geography node in Fig. 3C. **Table S5.2.** The conditional probability of the Economy status node in Fig. 3C. **Table S5.3.** The conditional probability of the HDI node in Fig. 3C. **Table S5.4.** The conditional probability of the Living Areas node in Fig. 3C. **Table S5.5.** The conditional probability of the MVPA node in Fig. 3C. **Table S6.1.** The conditional probability of the Geography node in Fig. 3D. **Table S6.2.** The conditional probability of the Economy status node in Fig. 3D. **Table S6.3.** The conditional probability of the HDI node in Fig. 3D. **Table S6.4.** The conditional probability of the Living Areas node in Fig. 3D. **Table S6.5.** The conditional probability of the Sedentary Behavior node in Fig. 3D. **Table S7.** Prevalence of different chronic diseases by province.

## Data Availability

The datasets analyzed during the current study are available from the corresponding author on reasonable request.

## Abbreviations

PA: Physical Activity
PI: Physical Inactivity
NCDs: Non-communicable diseases
CLASS: China Longitudinal Aging Social Survey
HDI: Human Development Index
NSRC: National Survey Research Center at Renmin University of China
CSSN: China Social Survey Network
PSUs: Primary sampling units
SSUs: Secondary sampling units
MPA: Moderate-intensity Physical Activities
VPA: Vigorous-intensity Physical Activity
LPA: Light-intensity Physical Activity
MET: Metabolic Equivalent
WHO: World Health Organization
IPAQ: International Physical Activity Questionnaire
MVPA: Moderate-to-Vigorous Physical Activity
LTPA: Leisure Time Physical Activity
GDP: Gross domestic production
BMI: Body Mass Index
