# Supplementary material for: Association of physical activity with socio-economic status and chronic disease in older adults in China: cross-sectional findings from the survey of CLASS 2020 after the outbreak of COVID-19
Source: BMC Public Health. 2024 Jan 2;24:37. doi: 10.1186/s12889-023-17492-9 (PMC10762973; doi:10.1186/s12889-023-17492-9)
Supplement: Supplementary file 1 — Additional file 1: Table S1. The basis for dividing the east areas and west areas. Table S2. Variables and their assignments in the Bayesian Network model. Table S3.1. The conditional probability of the Geography node in Fig. 3A. Table S3.2. The conditional probability of the Economy status node in Fig. 3A. Table S3.3. The conditional probability of the HDI node in Fig. 3A. Table S3.4. The conditional probability of the Living Areas node in Fig. 3A. Table S3.5. The conditional probability of the LPA node in Fig. 3A. Table S4.1. The conditional probability of the Geography node in Fig. 3B. Table S4.2. The conditional probability of the Economy status node in Fig. 3B. Table S4.3. The conditional probability of the HDI node in Fig. 3B. Table S4.4. The conditional probability of the Living Areas node in Fig. 3B. Table S4.5. The conditional probability of the MPA node in Fig. 3B. Table S5.1. The conditional probability of the Geography node in Fig. 3C. Table S5.2. The conditional probability of the Economy status node in Fig. 3C. Table S5.3. The conditional probability of the HDI node in Fig. 3C. Table S5.4. The conditional probability of the Living Areas node in Fig. 3C. Table S5.5. The conditional probability of the MVPA node in Fig. 3C. Table S6.1. The conditional probability of the Geography node in Fig. 3D. Table S6.2. The conditional probability of the Economy status node in Fig. 3D. Table S6.3. The conditional probability of the HDI node in Fig. 3D. Table S6.4. The conditional probability of the Living Areas node in Fig. 3D. Table S6.5. The conditional probability of the Sedentary Behavior node in Fig. 3D. Table S7. Prevalence of different chronic diseases by province. [file 12889_2023_17492_MOESM1_ESM.docx]

Supplementary appendix

Table S1 The basis for dividing the east areas and west areas

| Province Name | Is it crossed by the Heihe -Tengchong Line | Number of cities surveyed located on the left side of the Heihe Tengchong line | Number of cities surveyed located on the right side of the Heihe Tengchong line | Location of province |
| --- | --- | --- | --- | --- |
| Inner Mongolia | yes | 1 | 0 | west areas |
| Ningxia | no | \ | \ | west areas |
| Gansu | no | \ | \ | west areas |
| Shaanxi | yes | 2 | 1 | west areas |
| Qinghai | no | \ | \ | west areas |
| Heilongjiang | yes | 1 | 3 | east areas |
| Jilin | no | \ | \ | east areas |
| Liaoning | no | \ | \ | east areas |
| Beijing | no | \ | \ | east areas |
| Tianjin | no | \ | \ | east areas |
| Hebei | yes | 0 | 3 | east areas |
| Shanxi | yes | 0 | 3 | east areas |
| Sichuan | yes | 0 | 6 | east areas |
| Yunnan | yes | 0 | 4 | east areas |
| Shandong | no | \ | \ | east areas |
| Henan | no | \ | \ | east areas |
| Jiangsu | no | \ | \ | east areas |
| Shanghai | no | \ | \ | east areas |
| Zhejiang | no | \ | \ | east areas |
| Hunan | no | \ | \ | east areas |
| Hubei | no | \ | \ | east areas |
| Chongqing | no | \ | \ | east areas |
| Jiangxi | no | \ | \ | east areas |
| Guizhou | no | \ | \ | east areas |
| Guangdong | no | \ | \ | east areas |
| Guangxi | no | \ | \ | east areas |
| Fujian | no | \ | \ | east areas |

Table S2 Variables and their assignments in the Bayesian Network model.

| Factors | Assignment |
| --- | --- |
| Geography | West areas = 0; East areas = 1 |
| Economy status | Low GDP areas = 0; High GDP areas = 1 |
| HDI | ＜0.691 = 0; 0.728~= 1; ≥0.803 = 2 |
| Living Areas | Rural = 0；Urban = 1 |
| LPA (minutes) | 0 = 0; 10~30 = 1; 40~510 = 2; 520~1290 = 3; 1300~2700 = 4 |
| MPA (minutes) | 0 = 0; 10~90 = 1; 100~200 = 2; 210~360 = 3; 370~2100 = 4 |
| MVPA (minutes) | 0 = 0; 10~120 = 1; 130~210 = 2; 220~420 = 3; 425~2470 = 4 |
| Sedentary Behavior(hour) | 0 = 0; 1~16.5 = 1; 16.6~21 = 2; 21.1~28 = 3; 28.1~90 = 4 |

Table S3.1 The conditional probability of the Geography node in Figure 3A

| Factors | Value of conditional probability |
| --- | --- |
| Geography = 0 | 0.11068 |
| Geography = 1 | 0.88932 |

Table S3.2 The conditional probability of the Economy status node in Figure 3A

|  | Geography = 0 | Geography = 1 |
| --- | --- | --- |
| Economy status = 0 | 0.81778 | 0.54753 |
| Economy status = 1 | 0.18222 | 0.45247 |

Table S3.3 The conditional probability of the HDI node in Figure 3A

|  | Geography = 0  Economy status = 0 | Geography = 0  Economy status = 1 | Geography = 1  Economy status = 0 | Geography = 1  Economy status = 1 |
| --- | --- | --- | --- | --- |
| HDI = 0 | 0.33185 | 0.33333 | 0.13176 | 0.01671 |
| HDI = 1 | 0.59388 | 0.33333 | 0.85443 | 0.57605 |
| HDI = 2 | 0.07427 | 0.33333 | 0.01381 | 0.40724 |

Table S3.4 The conditional probability of the Living Areas node in Figure 3A

|  | Geography = 0  HDI = 0 | Geography = 0  HDI = 1 | Geography = 0  HDI = 2 | Geography = 1  HDI = 0 | Geography = 1  HDI = 1 | Geography = 1  HDI = 2 |
| --- | --- | --- | --- | --- | --- | --- |
| Living Areas = 0 | 0.54060 | 0.59671 | 0.50000 | 0.58080 | 0.49016 | 0.18931 |
| Living Areas = 1 | 0.45940 | 0.40329 | 0.50000 | 0.41920 | 0.50984 | 0.81069 |

Table S3.5 The conditional probability of the LPA node in Figure 3A

|  | Living Areas = 0  Geography = 0  Economy status = 0  HDI =0 | Living Areas = 0  Geography = 0  Economy status = 0  HDI =1 | Living Areas = 0  Geography = 0  Economy status = 0  HDI =2 | Living Areas = 0  Geography = 0  Economy status = 1  HDI =0 | Living Areas = 0  Geography = 0  Economy status = 1  HDI =1 | Living Areas = 0  Geography = 0  Economy status = 1  HDI =2 |
| --- | --- | --- | --- | --- | --- | --- |
| LPA (minutes) = 0 | 0.08958 | 0.03287 | 0.20000 | 0.20000 | 0.20000 | 0.20000 |
| LPA (minutes) = 1 | 0.05537 | 0.02794 | 0.20000 | 0.20000 | 0.20000 | 0.20000 |
| LPA (minutes) = 2 | 0.17264 | 0.19803 | 0.20000 | 0.20000 | 0.20000 | 0.20000 |
| LPA (minutes) = 3 | 0.31433 | 0.30649 | 0.20000 | 0.20000 | 0.20000 | 0.20000 |
| LPA (minutes) = 4 | 0.36808 | 0.43468 | 0.20000 | 0.20000 | 0.20000 | 0.20000 |
|  | Living Areas = 0  Geography = 1  Economy status = 0  HDI =0 | Living Areas = 0  Geography = 1  Economy status = 0  HDI =1 | Living Areas = 0  Geography = 1  Economy status = 0  HDI =2 | Living Areas = 0  Geography = 1  Economy status = 1  HDI =0 | Living Areas = 0  Geography = 1  Economy status = 1  HDI =1 | Living Areas = 0  Geography = 1  Economy status = 1  HDI =2 |
| LPA (minutes) = 0 | 0.70057 | 0.24968 | 0.20000 | 0.20000 | 0.41847 | 0.39684 |
| LPA (minutes) = 1 | 0.01991 | 0.01103 | 0.20000 | 0.20000 | 0.01487 | 0.02323 |
| LPA (minutes) = 2 | 0.06472 | 0.16247 | 0.20000 | 0.20000 | 0.24007 | 0.26301 |
| LPA (minutes) = 3 | 0.09673 | 0.24189 | 0.20000 | 0.20000 | 0.20132 | 0.22398 |
| LPA (minutes) = 4 | 0.11807 | 0.33493 | 0.20000 | 0.20000 | 0.12527 | 0.09294 |
|  | Living Areas = 1  Geography = 0  Economy status = 0  HDI =0 | Living Areas = 1  Geography = 0  Economy status = 0  HDI =1 | Living Areas = 1  Geography = 0  Economy status = 0  HDI =2 | Living Areas = 1  Geography = 0  Economy status = 1  HDI =0 | Living Areas = 1  Geography = 0  Economy status = 1  HDI =1 | Living Areas = 1  Geography = 0  Economy status = 1  HDI =2 |
| LPA (minutes) = 0 | 0.08549 | 0.03581 | 0.20000 | 0.20000 | 0.20000 | 0.20000 |
| LPA (minutes) = 1 | 0.06163 | 0.04348 | 0.20000 | 0.20000 | 0.20000 | 0.20000 |
| LPA (minutes) = 2 | 0.22266 | 0.15857 | 0.20000 | 0.20000 | 0.20000 | 0.20000 |
| LPA (minutes) = 3 | 0.30616 | 0.32737 | 0.20000 | 0.20000 | 0.20000 | 0.20000 |
| LPA (minutes) = 4 | 0.32406 | 0.43478 | 0.20000 | 0.20000 | 0.20000 | 0.20000 |
|  | Living Areas = 1  Geography = 1  Economy status = 0  HDI =0 | Living Areas = 1  Geography = 1  Economy status = 0  HDI =1 | Living Areas =1  Geography = 1  Economy status = 0  HDI =2 | Living Areas = 1  Geography = 1  Economy status = 1  HDI =0 | Living Areas = 1  Geography = 1  Economy status = 1  HDI =1 | Living Areas = 1  Geography = 1  Economy status = 1  HDI =2 |
| LPA (minutes) = 0 | 0.29490 | 0.18439 | 0.20000 | 0.20000 | 0.25659 | 0.11499 |
| LPA (minutes) = 1 | 0.02857 | 0.00863 | 0.20000 | 0.20000 | 0.00886 | 0.01455 |
| LPA (minutes) = 2 | 0.16020 | 0.16160 | 0.20000 | 0.20000 | 0.47044 | 0.40913 |
| LPA (minutes) = 3 | 0.21531 | 0.28290 | 0.20000 | 0.20000 | 0.20611 | 0.32603 |
| LPA (minutes) = 4 | 0.30102 | 0.36248 | 0.20000 | 0.20000 | 0.05800 | 0.13531 |

Table S4.1 The conditional probability of the Geography node in Figure 3B

| Factors | Value of conditional probability |
| --- | --- |
| Geography = 0 | 0.11068 |
| Geography = 1 | 0.88932 |

Table S4.2 The conditional probability of the Economy status node in Figure 3B

|  | Geography = 0 | Geography = 1 |
| --- | --- | --- |
| Economy status = 0 | 0.81778 | 0.54753 |
| Economy status = 1 | 0.18222 | 0.45247 |

Table S4.3 The conditional probability of the HDI node in Figure 3B

|  | Geography = 0  Economy status = 0 | Geography = 0  Economy status = 1 | Geography = 1  Economy status = 0 | Geography = 1  Economy status = 1 |
| --- | --- | --- | --- | --- |
| HDI = 0 | 0.33185 | 0.33333 | 0.13176 | 0.01671 |
| HDI = 1 | 0.59388 | 0.33333 | 0.85443 | 0.57605 |
| HDI = 2 | 0.07427 | 0.33333 | 0.01381 | 0.40724 |

Table S4.4 The conditional probability of the Living Areas node in Figure 3B

|  | Geography = 0  HDI = 0 | Geography = 0  HDI = 1 | Geography = 0  HDI = 2 | Geography = 1  HDI = 0 | Geography = 1  HDI = 1 | Geography = 1  HDI = 2 |
| --- | --- | --- | --- | --- | --- | --- |
| Living Areas = 0 | 0.54060 | 0.59671 | 0.50000 | 0.58080 | 0.49016 | 0.18931 |
| Living Areas = 1 | 0.45940 | 0.40329 | 0.50000 | 0.41920 | 0.50984 | 0.81069 |

Table S4.5 The conditional probability of the MPA node in Figure 3B

|  | Living Areas = 0  Economy status = 0  HDI =0 | Living Areas = 0  Economy status = 0  HDI =1 | Living Areas = 0  Economy status = 0  HDI =2 | Living Areas = 0  Economy status = 1  HDI =0 | Living Areas = 0  Economy status = 1  HDI =1 | Living Areas = 0  Economy status = 1  HDI =2 |
| --- | --- | --- | --- | --- | --- | --- |
| MPA (minutes) = 0 | 0.85347 | 0.93321 | 0.20000 | 0.20000 | 0.87961 | 0.67111 |
| MPA (minutes) = 1 | 0.04851 | 0.03082 | 0.20000 | 0.20000 | 0.02318 | 0.04663 |
| MPA (minutes) = 2 | 0.03218 | 0.01266 | 0.20000 | 0.20000 | 0.03311 | 0.06162 |
| MPA (minutes) = 3 | 0.03812 | 0.01199 | 0.20000 | 0.20000 | 0.03240 | 0.10408 |
| MPA (minutes) = 4 | 0.02772 | 0.01132 | 0.20000 | 0.20000 | 0.03169 | 0.11657 |
|  | Living Areas = 1  Economy status = 0  HDI =0 | Living Areas = 1  Economy status = 0  HDI =1 | Living Areas =1  Economy status = 0  HDI =2 | Living Areas = 1  Econom  status = 1  HDI =0 | Living Areas = 1  Economy status = 1  HDI =1 | Living Areas = 1  Economy status = 1  HDI =2 |
| MPA (minutes) = 0 | 0.77006 | 0.86254 | 0.20000 | 0.20000 | 0.72167 | 0.57612 |
| MPA (minutes) = 1 | 0.10047 | 0.05498 | 0.20000 | 0.20000 | 0.03274 | 0.09547 |
| MPA (minutes) = 2 | 0.04181 | 0.02866 | 0.20000 | 0.20000 | 0.12452 | 0.07330 |
| MPA (minutes) = 3 | 0.04383 | 0.02796 | 0.20000 | 0.20000 | 0.07798 | 0.11705 |
| MPA (minutes) = 4 | 0.04383 | 0.02585 | 0.20000 | 0.20000 | 0.04308 | 0.13805 |

Table S5.1 The conditional probability of the Geography node in Figure 3C

| Factors | Value of conditional probability |
| --- | --- |
| Geography = 0 | 0.11068 |
| Geography = 1 | 0.88932 |

Table S5.2 The conditional probability of the Economy status node in Figure 3C

|  | Geography = 0 | Geography = 1 |
| --- | --- | --- |
| Economy status = 0 | 0.81778 | 0.54753 |
| Economy status = 1 | 0.18222 | 0.45247 |

Table S5.3 The conditional probability of the HDI node in Figure 3C

|  | Geography = 0  Economy status = 0 | Geography = 0  Economy status = 1 | Geography = 1  Economy status = 0 | Geography = 1  Economy status = 1 |
| --- | --- | --- | --- | --- |
| HDI = 0 | 0.33185 | 0.33333 | 0.13176 | 0.01671 |
| HDI = 1 | 0.59388 | 0.33333 | 0.85443 | 0.57605 |
| HDI = 2 | 0.07427 | 0.33333 | 0.01381 | 0.40724 |

Table S5.4 The conditional probability of the Living Areas node in Figure 3C

|  | Geography = 0  HDI = 0 | Geography = 0  HDI = 1 | Geography = 0  HDI = 2 | Geography = 1  HDI = 0 | Geography = 1  HDI = 1 | Geography = 1  HDI = 2 |
| --- | --- | --- | --- | --- | --- | --- |
| Living Areas = 0 | 0.54060 | 0.59671 | 0.50000 | 0.58080 | 0.49016 | 0.18931 |
| Living Areas = 1 | 0.45940 | 0.40329 | 0.50000 | 0.41920 | 0.50984 | 0.81069 |

Table S5.5 The conditional probability of the MVPA node in Figure 3C

|  | Living Areas = 0  Economy status = 0  HDI =0 | Living Areas = 0  Economy status = 0  HDI =1 | Living Areas = 0  Economy status = 0  HDI =2 | Living Areas = 0  Economy status = 1  HDI =0 | Living Areas = 0  Economy status = 1  HDI =1 | Living Areas = 0  Economy status = 1  HDI =2 |
| --- | --- | --- | --- | --- | --- | --- |
| MVPA (minutes) = 0 | 0.85198 | 0.93052 | 0.20000 | 0.20000 | 0.87677 | 0.67111 |
| MVPA (minutes) = 1 | 0.04554 | 0.02947 | 0.20000 | 0.20000 | 0.03098 | 0.05412 |
| MVPA (minutes) = 2 | 0.03366 | 0.01434 | 0.20000 | 0.20000 | 0.02673 | 0.05412 |
| MVPA (minutes) = 3 | 0.04109 | 0.01132 | 0.20000 | 0.20000 | 0.03524 | 0.11157 |
| MVPA (minutes) = 4 | 0.02772 | 0.01434 | 0.20000 | 0.20000 | 0.03027 | 0.10908 |
|  | Living Areas = 1  Economy status = 0  HDI =0 | Living Areas = 1  Economy status = 0  HDI =1 | Living Areas =1  Economy status = 0  HDI =2 | Living Areas = 1  Economy status = 1  HDI =0 | Living Areas = 1  Economy status = 1  HDI =1 | Living Areas = 1  Economy status = 1  HDI =2 |
| MVPA (minutes) = 0 | 0.76601 | 0.85412 | 0.20000 | 0.20000 | 0.85412 | 0.57437 |
| MVPA (minutes) = 1 | 0.10452 | 0.04902 | 0.20000 | 0.20000 | 0.06894 | 0.11939 |
| MVPA (minutes) = 2 | 0.04181 | 0.04181 | 0.20000 | 0.20000 | 0.10319 | 0.04880 |
| MVPA (minutes) = 3 | 0.04990 | 0.03007 | 0.20000 | 0.20000 | 0.08768 | 0.13047 |
| MVPA (minutes) = 4 | 0.13047 | 0.03463 | 0.20000 | 0.20000 | 0.02047 | 0.12697 |

Table S6.1 The conditional probability of the Geography node in Figure 3D

| Factors | Value of conditional probability |
| --- | --- |
| Geography = 0 | 0.11068 |
| Geography = 1 | 0.88932 |

Table S6.2 The conditional probability of the Economy status node in Figure 3D

|  | Geography = 0 | Geography = 1 |
| --- | --- | --- |
| Economy status = 0 | 0.81778 | 0.54753 |
| Economy status = 1 | 0.18222 | 0.45247 |

Table S6.3 The conditional probability of the HDI node in Figure 3D

|  | Geography = 0  Economy status = 0 | Geography = 0  Economy status = 1 | Geography = 1  Economy status = 0 | Geography = 1  Economy status = 1 |
| --- | --- | --- | --- | --- |
| HDI = 0 | 0.33185 | 0.33333 | 0.13176 | 0.01671 |
| HDI = 1 | 0.59388 | 0.33333 | 0.85443 | 0.57605 |
| HDI = 2 | 0.07427 | 0.33333 | 0.01381 | 0.40724 |

Table S6.4 The conditional probability of the Living Areas node in Figure 3D

|  | Geography = 0  HDI = 0 | Geography = 0  HDI = 1 | Geography = 0  HDI = 2 | Geography = 1  HDI = 0 | Geography = 1  HDI = 1 | Geography = 1  HDI = 2 |
| --- | --- | --- | --- | --- | --- | --- |
| Living Areas = 0 | 0.54060 | 0.59671 | 0.50000 | 0.58080 | 0.49016 | 0.18931 |
| Living Areas = 1 | 0.45940 | 0.40329 | 0.50000 | 0.41920 | 0.50984 | 0.81069 |

Table S6.5 The conditional probability of the Sedentary Behavior node in Figure 3D

|  | Living Areas = 0  Economy status = 0 | Living Areas = 0  Economy status = 1 | Living Areas = 1  Economy status = 0 | Living Areas = 1  Economy status = 1 |
| --- | --- | --- | --- | --- |
| Sedentary Behavior(hour) = 0 | 0.01394 | 0.02641 | 0.01459 | 0.01494 |
| Sedentary Behavior(hour) = 1 | 0.29295 | 0.18384 | 0.25912 | 0.22333 |
| Sedentary Behavior(hour) = 2 | 0.44975 | 0.19810 | 0.48147 | 0.21136 |
| Sedentary Behavior(hour) = 3 | 0.13321 | 0.16059 | 0.13277 | 0.22870 |
| Sedentary Behavior(hour) = 4 | 0.11015 | 0.43106 | 0.11205 | 0.32167 |

Table S7 Prevalence of different chronic diseases by province

| Province Name | Diagnostic rates of heart disease | Diagnostic rates of cerebrovascular disease | Diagnostic rates of lumbar and cervical spondylosis | Diagnostic rates of arthritis or rheumatism | Diagnostic rates of respiratory diseases |
| --- | --- | --- | --- | --- | --- |
| National Average | 14.77% | 7.27% | 19.32% | 25.56% | 7.64% |
| Inner Mongolia | 21.70% | 8.49% | 4.71% | 12.26% | 3.77% |
| Ningxia | 8.25% | 10.31% | 3.09% | 35.05% | 2.06% |
| Gansu | 11.79% | 5.64% | 13.33% | 20.51% | 3.08% |
| Shaanxi | 13.95% | 12.89% | 11.05% | 10.26% | 7.37% |
| Qinghai | 9.57% | 2.12% | 11.70% | 12.77% | 0% |
| Heilongjiang | 15.74% | 7.38% | 5.57% | 12.62% | 2.79% |
| Jilin | 25.93% | 13.01% | 12.23% | 14.95% | 4.08% |
| Liaoning | 25.12% | 9.11% | 13.05% | 14.78% | 1.97% |
| Beijing | 21.08% | 6.16% | 39.18% | 32.46% | 15.30% |
| Tianjin | 7.11% | 3.42% | 18.42% | 15.53% | 7.63% |
| Hebei | 21.60% | 10.10% | 3.83% | 5.57% | 3.48% |
| Shanxi | 13.25% | 12.85% | 6.83% | 4.42% | 2.41% |
| Sichuan | 13.22% | 6.09% | 32.52% | 49.22% | 9.57% |
| Yunnan | 16.83% | 7.56% | 39.02% | 52.93% | 19.02% |
| Shandong | 9.11% | 6.95% | 22.02% | 22.85% | 8.28% |
| Henan | 20.60% | 8.24% | 26.62% | 33.12% | 8.72% |
| Jiangsu | 11.38% | 4.27% | 15.85% | 21.14% | 8.13% |
| Shanghai | 24.81% | 7.78% | 21.85% | 21.30% | 7.96% |
| Zhejiang | 8.89% | 7.59% | 12.15% | 16.27% | 8.46% |
| Hunan | 17.72% | 15.68% | 42.57% | 50.31% | 11.41% |
| Hubei | 14.36% | 9.18% | 17.53% | 22.70% | 12.35% |
| Chongqing | 11.41% | 3.36% | 15.10% | 26.85% | 6.04% |
| Jiangxi | 2.65% | 1.13% | 7.37% | 7.37% | 1.13% |
| Guizhou | 6.62% | 6.62% | 4.97% | 12.25% | 3.64% |
| Guangdong | 8.81% | 2.87% | 36.97% | 58.24% | 11.88% |
| Guangxi | 3.67% | 2.82% | 13.84% | 34.75% | 1.98% |
| Fujian | 14.05% | 5.88% | 23.53% | 26.47% | 17.32% |
